# Adaptive Bacteria Colony Picking in Unstructured Environments Using Intensity Histogram and Unascertained LS-SVM Classifier

**DOI:** 10.1155/2014/928395

**Published:** 2014-05-12

**Authors:** Kun Zhang, Minrui Fei, Xin Li, Huiyu Zhou

**Affiliations:** ^1^School of Mechatronic Engineering & Automation, Shanghai University, M8 Building, 149 Yanchang Road, ZhaBei District, Shanghai 200072, China; ^2^The Institute of Electronics, Communications and Information Technology, Queen's University Belfast, Belfast, UK

## Abstract

Features analysis is an important task which can significantly affect the performance of automatic bacteria colony picking. Unstructured environments also affect the automatic colony screening. This paper presents a novel approach for adaptive colony segmentation in unstructured environments by treating the detected peaks of intensity histograms as a morphological feature of images. In order to avoid disturbing peaks, an entropy based mean shift filter is introduced to smooth images as a preprocessing step. The relevance and importance of these features can be determined in an improved support vector machine classifier using unascertained least square estimation. Experimental results show that the proposed unascertained least square support vector machine (ULSSVM) has better recognition accuracy than the other state-of-the-art techniques, and its training process takes less time than most of the traditional approaches presented in this paper.

## 1. Introduction

**Figure 1:**
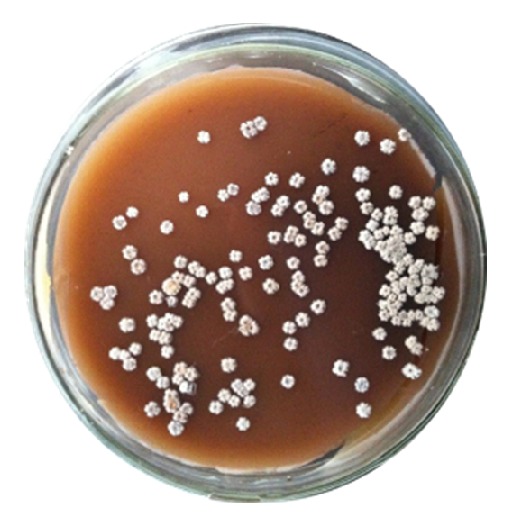
Erythrosin bacteria colony on agar.

**Figure 2:**
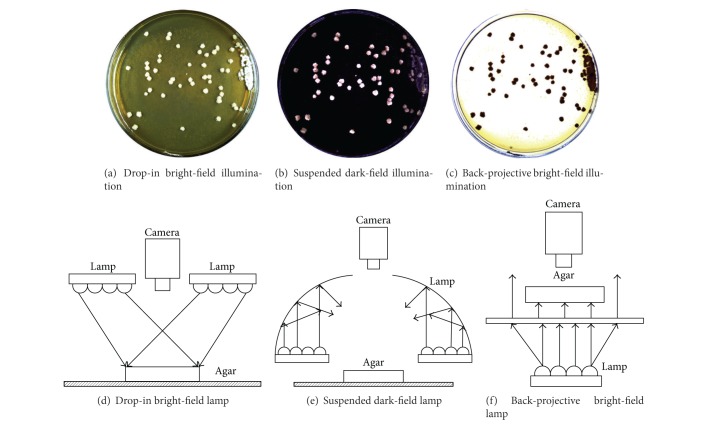
Three illumination technologies.

Bacteria colony isolation [1] is a labor intensive task over the past decades. Manual bacteria colony picking is tedious and experience dependent. Colony screening is in unstructured environments due to different agar mediums and cultivations.  Figure 1 is an example of Erythrosin bacteria colony sitting on agar. An automatic colony picking system can be used to make this process consistent and reliable with less time consumption. Researchers worldwide are currently seeking fast and reliable methods for high throughput colony picking. To achieve this, we need to make sure high quality colony illumination and image segmentation are the critical stages of a colony picking system. Currently, there are three major illumination techniques used for image acquisition: (1) drop-in bright-field illumination, (2) back-projective bright-field illumination, and (3) suspended dark-field illumination.  Figure 2 shows the different imaging quality based on the three techniques introduced above. Suspended dark-filed illumination based approaches can be used to reduce the influence of lights. In this circumstance, colony agar plates are placed in a suspended dark-field environment. Using reflected and refractive lights, we can obtain volumetric structures of colonies with good image quality. However, the suspended dark-filed illumination based approaches could achieve less satisfactory quality of images than the other two approaches, due to a similar color caused by the crowdedness of colonies. Image segmentation approaches such as thresholding [2], region growing [3], watershed [4], and mean shift [5] are commonly used in medical image analysis. Each of these classical methods has its own strengths and weaknesses. For example, a thresholding method is fast but requires the systematic parameters to be changed for different environments. Region growing methods are more robust than the thresholding methods but lack sufficient efficiency. The colony picking systems available on the market have a number of specific requirements in order to achieve good segmentation performance, for example, setting the region of interests, controlling the extent of cluttering, and maintaining appropriate light conditions.

In this paper, we deploy an intensity histogram based morphological features extraction algorithm, which contributes to colony analysis. The proposed method employs a peak-searching method in a standard intensity histogram. Afterwards, to achieve correct colony feature classification, we propose an entropy based mean shift algorithm to smooth the image as a preprocess stage. Finally, we introduce an improved approach for feature selection using an unascertained least square support vector machine (ULSSVM) classifier. To our knowledge, this is the first attempt to use the unascertained attributes of the detected features for the purpose of classification. We evaluate the proposed approach on a large dataset of colony images. Based on these experiments, we show that our approach can efficiently deliver correct classification results.

## 2. Proposed Approach

In the smoothing process of image, it is very important for a nonlinear filter to keep the fringe detail of an image during the process. Optimization techniques have been popularly used in image processing. This is driven by the performance need as the target of an application. However, it is very difficult to obtain an optimal solution (stopping criterion) for individual applications. More details can be found in [6–9]. Mean shift has proven to be appliance effective tool for image processing because of its nonparametric property. Smoothing by mean shift algorithm has been reported in the literature. For example, in [10], Zhao and Xi introduced mean shift as a smooth filter for processing YIQ color images and compared it with Wiener filter. In [11], Han and Sohn used mean shift combined with a sigma filter in an illumination and color compensation system. In literature [12], Sahba and Venetsanopoulos applied mean shift to reserve fringe detail and detect breast mass. These results are promising but the computational speed of mean shift is unexpectedly slow.

Entropy is a measure of complexity. We can also use entropy to inspect system uncertainty. Low entropy images have very little contrast and large runs of pixels with the same value. An image that is perfectly flat will have entropy of zero. Consequently, they can be compressed to a relatively small size. On the other hand, high entropy images have a great deal of contrast from one pixel to the next and consequently have more details than low entropy images. Entropy has been applied in pattern recognition, object tracking, and image segmentation, for example, [13–15], where entropy has been used as a termination criterion. As mentioned in the first section, the proposed feature based colony classification approach begins with entropy based mean shift filter, and it is followed by applying intensity histogram analysis to the filtered images. The characteristic peaks' coefficients retrieved from intensity histograms are then applied to colony classification within the framework of unascertained least square support vector machine.

### 2.1. Entropy Based Mean Shift Filter

The following is the idea of a standard mean shift approach [16]. Let *x*_*j*_ be a numerical sample of *n* in a *d*-dimensional space. The basic mean shift is defined as

(1) $$\begin{array}{c} M_{h}\left(x\right)=\frac{1}{k}\underset{x_{i}\in g_{h}}{\sum }\left(x_{i}-x\right), \end{array}$$

where *g*_*h*_ is a window based on the center *x* and radius *h*. *k* is the sample set number in *g*_*h*_. (*x*_*i*_ − *x*) is the relative offset of center *x*.

Equation (1) is a monotonic form and less effective in a practical application. A Kernel based mean shift algorithm is described as follows:

(2) $$\begin{array}{c} M_{h}\left(x\right)=\frac{\sum_{i=1}^{n}Q\left(\left(x_{i}-x\right)/h\right)\alpha \left(x_{i}-x\right)}{\sum_{i=1}^{n}Q\left(\left(x_{i}-x\right)/h\right)\alpha \left(x_{i}\right)}, \end{array}$$

where *α*(*x*) is the self-impact factor and *Q*(*x*) is a kernel function.

In a color image with *n* × *n* pixels, each pixel corresponds to a 5-dimension vector *R* ^5^ (*R*, *G*, *B*, *X*, *Y*). Due to the independence of space and color information, the kernel function is obtained in

(3) $$\begin{array}{c} Q_{g_{s}g_{r}}\left(x\right)=\frac{1}{g_{s}^{2}g_{r}^{3}}q\left({||\frac{x^{s}}{g_{s}}||}^{2}\right)q\left({||\frac{x^{r}}{g_{r}}||}^{2}\right), \end{array}$$

where *x* ^*s*^ is the spatial position of a pixel; *x* ^*r*^ is the color information of a pixel; *g*_*s*_ is a spatial window based on the center *x* and radius *s*; *g*_*r*_ is a color window based on the center *x* and radius *r*.

Let *q*(*x*_*t*_) be the gray value probability of the outcome *x*_*t*_, *k* = 1,…*n*. *A* is an image with log_2_(0) = 0; log_2_(1/*q*(*x*_*t*_)) is called the surprisal of the outcome *x*_*t*_. Entropy is defined as

(4) $$\begin{array}{c} F\left(X\right)\equiv \overset{2^{n}}{\underset{t=1}{\sum }}q\left(x_{t}\right)\text{lo}{\text{g}}_{2}\left(\frac{1}{q\left(x_{t}\right)}\right)=-\overset{2^{n}}{\underset{t=1}{\sum }}q\left(x_{t}\right)\text{lo}{\text{g}}_{2}q\left(x_{t}\right). \end{array}$$

Entropy is determined based on the pixels distribution in an image, which is influenced by two factors: foreground and background or called noise. The uncertainty of entropy is dominated by the noise's variance. Entropy can be used to measure the homogeneity of an image area: the more homogeneous image, the less the entropy values. In practice, when we work with images, due to the noise, entropy cannot decrease to zero but it can reach a stable value. Thus, entropy can be applied as a stopping criterion for a mean shift iteration (Algorithm 1).

### 2.2. Model of Peak Searching in Intensity Histogram

Intensity histogram is an important feature of images and can be regarded as the approximate expression of a density function of image intensities. It shows the frequency of an intensity appearing in an image. An intensity histogram is described in

(5) $$\begin{array}{c} G_{i}=\overset{M}{\underset{m=1}{\sum }}\;\overset{N}{\underset{n=1}{\sum }}P\left(i,m,n\right), \end{array}$$

where *M* and *N* represent the total numbers of rows and columns, respectively, and *G*_*i*_ represents the appearance times of intensity *i*, and *P*(*i*, *m*, *n*) is described as follows:

(6) $$\begin{array}{c} P\left(i,m,n\right)=\{\begin{array}{cc} 1 & P\left(m,n\right)=i\\ 0 & P\left(m,n\right)\neq i, \end{array} \end{array}$$

where *P*(*m*, *n*) is the intensity value of point (*m*, *n*). As the sizes of different images may be different, to avoid the impact of image size, we normalize each image according to the following equation:

(7) $$\begin{array}{c} G_{i}^{\prime }=\frac{G_{i}}{T}, \end{array}$$

where *G*_*i*_′ is a normalization value and *T* represents the total amount of the pixels of an image.  Figure 1 shows an example of bacteria colony and its intensity histogram using (5), (6), and (7).

**Figure 3:**
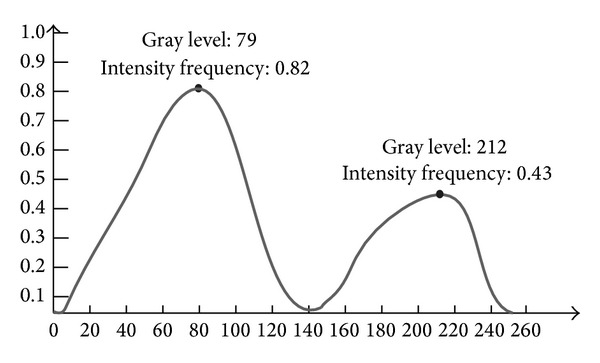
Normalised intensity histogram.

In Figure 1, the entire image can be divided into two main zones: culture medium zone and colony zone, according to the contrast and density of the image. In Figure 3, there are two peaks, which represent high frequency values of the corresponding intensities. The difference between the gray levels of the two peaks is 133, and the difference between the two intensity frequencies of the two peaks is 0.39. The intensity histogram mathematical model will be introduced in the following sections.

The peaks and valleys shown in Figure 3 can be obtained using the second-order derivative. The method to find a peak or a valley is described in

(8) $$\begin{array}{c} \Delta G_{i}^{\prime }=\{\begin{array}{cc} 1 & G_{i}^{\prime }-G_{i+1}^{\prime }<-H\\ 0 & \left|G_{i}^{\prime }-G_{i+1}^{\prime }\right|<H\\ -1 & G_{i}^{\prime }-G_{i+1}^{\prime }>H, \end{array} \end{array}$$

where *H* refers to a positive threshold which is set according to a specific image to reduce inaccuracy because of infinitesimal disturbance. Δ*G*_*i*_′ represents the tendency of the histogram curve at the point where the intensity equals *i*. Δ*G*_*i*_′ = 1 means that the curve ascends, Δ*G*_*i*_′ = −1 means that the curve descends, and Δ*G*_*i*_′ = 0 means that the curve is flat. Therefore, 1(0 ⋯ 0) − 1 indicates the peak of the curve, and −1(0 ⋯ 0)1 refers to the valley of the curve. In Figure 3, the peaks have been extracted according to the method described above.

Through the experiments, it is found that most of the intensity histogram curves change from double-peak to multipeak due to different bacteria colony in different illumination conditions. It is possible to obtain multiple local peaks or valleys in case the boundary of the intensity histogram is not smooth. The proposed peaks searching algorithm is shown in Algorithm 2.

## 3. Unascertained Least Square Support Vector Machine

Support vector machines (SVM) have been well studied in the machine learning field, which was proposed by Vapnik [17]. The performance of SVM has been verified in many applications, such as handwriting recognition [18], face recognition [19], and medical pattern matching [20]. But the training speed of SVM is too slow and this hinders its applications. Different from the classical support vector machine methods, the least squares support vector machines (LSSVM) proposed by Suykens and Vandewalle [21] were to change the form of the original convex quadratic optimization problem into a linear optimization problem and they effectively enhance the training speed. But it is hard to classify some uncertain information. Based on LSSVM and unascertained mathematical models, we propose the ULSSVM algorithm. For unascertained information, we can use unascertained number [22] and unascertained programming [23] to describe our algorithm. Please see below for a summary of these theories.

**Theorem 1**

[*α*, *β*], *α* = *x*_1_ < *x*_2_ < ⋯, *x*_*n*_ = *β*, if function *f*(*x*) satisfies

(9)  f(x)={ϕix=xi,  i=1,…,l0x≠xi,  x∈[α,β], s.t. ∑i=1lϕi=ϕ, 0<ϕi≤1  (i=1,…,l),

where [*α*, *β*] and *f*(*x*) form *l* order unascertained numbers and are set as [[*α*, *β*], *f*(*x*)]. *ϕ* is the main reliability. [*α*, *β*] is the value range. *f*(*x*) is the main reliability distribution density function. {*x*_*i*_} is a possible value sequence of the unascertained numbers. {*ϕ*_*i*_} is a confidence value sequence of the unascertained numbers.

**Theorem 2**

Setting the unascertained number *a* = [[*α*_1_, *α*_*n*_], *f*(*y*)], *b* = [[*β*_1_, *β*_*m*_], *k*(*x*)],

(10) $$\begin{array}{c} f\left(y\right)=\{\begin{array}{cc} \phi_{i}, & y=y_{i},\;\;i=1,\ldots ,l\\ 0, & y\neq y_{i},\;\;y\in \left[y_{1},y_{l}\right], \end{array} \end{array}$$

(11) $$\begin{array}{c} \overset{l}{\underset{i=1}{\sum }}\phi_{i}=\phi ,\;0<\phi_{i}\leq 1\left(i=1,\ldots ,l\right), \end{array}$$

(12) $$\begin{array}{c} k\left(x\right)=\{\begin{array}{cc} \gamma_{j}, & x=x_{j},\;\;j=1,\ldots ,p\\ 0, & x\neq x_{j},\;\;x\in \left[x_{1},x_{p}\right], \end{array} \end{array}$$

(13) $$\begin{array}{c} \overset{P}{\underset{j=1}{\sum }}\gamma_{j}=\gamma ,\;0<\gamma_{j}\leq 1\left(j=1,\ldots ,p\right). \end{array}$$

One calls inequalities *a* ≤ *b*, *b* ≤ *a* unascertained events.

**Theorem 3**

One calls the following programing as an unascertained constraint programing:

(14) max⁡ f¯ s.t.    Cr{f(X,a)≥f¯}≥γ     Cr{gj(X,bj)≤0,j=1,…,p}≥ϕ,

where *X* is a decision vector and *a*, *b*_*j*_  (*j* = 1,…, *p*) are unascertained parameter vectors. *f*(*X*, *a*) is the target function. *g*_*j*_(*X*, *b*_*j*_) is a constraint function. *ϕ*, *γ*  (*ϕ*, *γ* ∈ (0,1])  are confidence levels of the constraint and target function. *Cr*{·} is a credible degree of the unascertained events.

Based on the preliminary knowledge mentioned above, if the SVM training data obtains unascertained information, we can transform the unascertained information into unascertained number

(15) $$\begin{array}{c} a=\left[\left[x_{1},x_{n}\right],f\left(x\right)\right],\\ f\left(x\right)=\{\begin{array}{cc} \phi_{j} & x=x_{j},\;\;j=1,\ldots ,l\\ 0 & x\neq x_{j},\;\;x\in \left[x_{1},x_{l}\right]. \end{array} \end{array}$$

The training set is defined in

(16) $$\begin{array}{c} K=\left\{\left(y_{1},a_{1}\right),\left(y_{2},a_{2}\right),\ldots ,\left(y_{l},a_{l}\right)\right\}, \end{array}$$

where *y*_*i*_ ∈ *R* ^*l*^, *a*_*i*_ is an unascertained number, (*y*_*i*_, *a*_*i*_)  (*i* = 1,…, *l*) is an unascertained training point, and *K* is an unascertained training set.

The objective function can be minimized as follows:

(17) min⁡ϖ,b,ξ J(w,ξ)=12||w||2+12γ∑i=1nξi2,s.t.   Ai[wTϕ(xi)+b]=1−ξi, i=1,…n.

We then define a Lagrange function as

(18) L(w,b,ξ,α)=12||w||2+12γ∑i=1nξi2 −∑i=1nαi{Ai[wTϕ(xi)+b]−1+ξi}.

According to the KKT condition,

(19) ∂L∂w=0⟹w=∑i=1nαiAiφ(xi),∂L∂b=0⟹∑i=1nαiAi=0,∂L∂ξi=0⟹αi=γξi,∂L∂αi=0⟹Ai[wTϕ(xi)+b]−1+ξi=0.

Equation (19) can turn into the following matrix problem:

(20) $$\begin{array}{c} \left[\begin{array}{cccc} L & 0 & 0 & -Z^{T}\\ 0 & 0 & 0 & -Y^{T}\\ 0 & 0 & \gamma^{I} & -L\\ Z & Y & L & 0 \end{array}\right]\left[\begin{array}{c} \varpi \\ b\\ \xi \\ \alpha \end{array}\right]=\left[\begin{array}{c} 0\\ 0\\ 0\\ L \end{array}\right], \end{array}$$

where *Z* = [*φ*(*x*_1_)^*T*^ *y*_1_, *φ*(*x*_2_)^*T*^ *y*_2_,…*φ*(*x*_*n*_)^*T*^ *y*_*n*_]^*T*^, *Y* = [*y*_1_, *y*_2_,…, *y*_*n*_], *L* = [1,1,…, 1]^*T*^, *ξ* = [*ξ*_1_, *ξ*_2_,…, *ξ*_*n*_], and *α* = [*α*_1_, *α*_2_,…, *α*_*n*_].

We eliminate *w* and *ξ* and then get the following equations:

(21) $$\begin{array}{c} \left[\begin{array}{cc} 0 & -Y^{T}\\ Y & \Omega +\frac{1}{\gamma }I \end{array}\right]\left[\begin{array}{c} b\\ \alpha \end{array}\right]=\left[\begin{array}{c} 0\\ L \end{array}\right], \end{array}$$

where *Ω* = *ZZ* ^*T*^,

(22) $$\begin{array}{c} \Omega_{kl}=y_{k}y_{l}\phi {\left(x_{k}\right)}^{T}\phi \left(x_{l}\right)=y_{k}y_{l}\psi \left(x_{k},x_{l}\right), \end{array}$$

where *Ω*(·) is a kernel function and satisfies the Mercer theorem.

A set of linear equations will be solved instead of a QP problem. Finally, we can obtain the following optimal classification function:

(23) $$\begin{array}{c} f\left(x\right)=\mathrm{sign}\left(\overset{l}{\underset{i=1}{\sum }}A_{i}\alpha_{i}^{*}\left(x\cdot x_{i}\right)+b^{*}\right), \end{array}$$

where *α*_i_* is the optimal solutions and corresponding bias b*. *A*_*i*_ is an unascertained set.

## 4. Experimental Results

The proposed algorithm is evaluated on colony image databases which are captured using a Basler CCD sensor. The images are resized to be 640 × 480. The used computer is of a 3.2 G CPU running Windows 7 with a 4 G memory. The first three experiments have been carried out using the colony images to analyze the feasibility and efficiency of the proposed algorithm with MATLAB 7.2. The last experiment is carried out to demonstrate the segmentation effect and performed with Visual Studio 2010.

**Figure 4:**
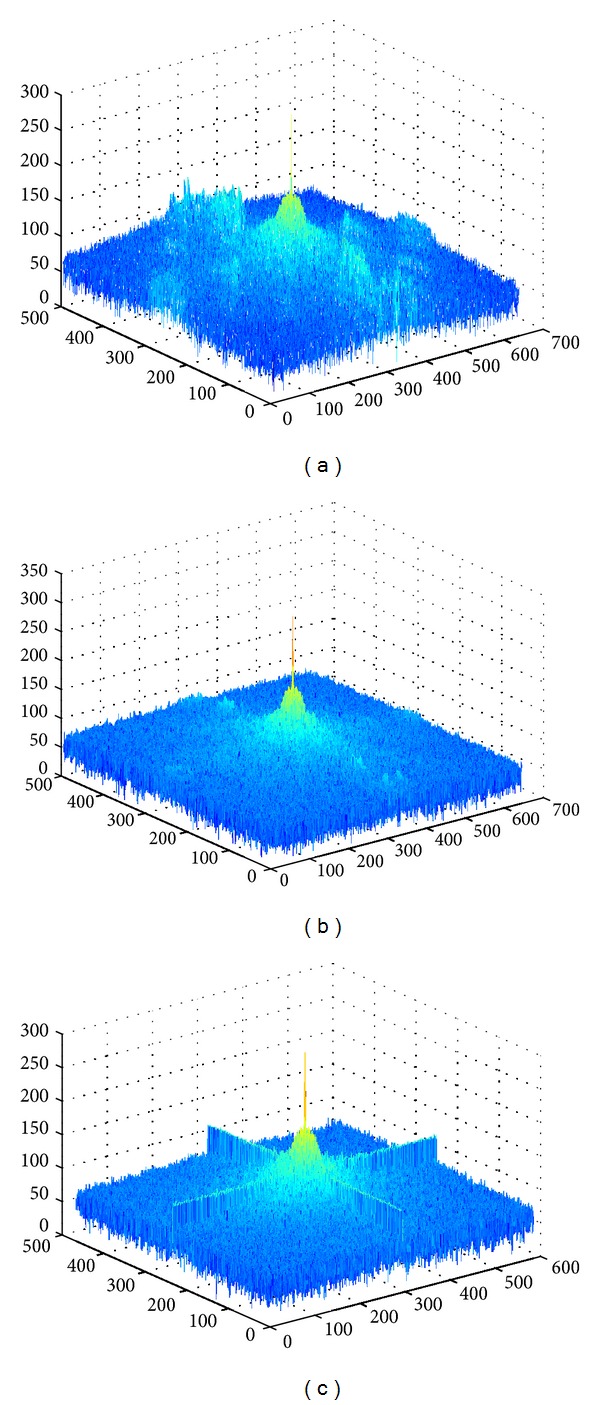
Energy spectrum at different stages.

**Figure 5:**
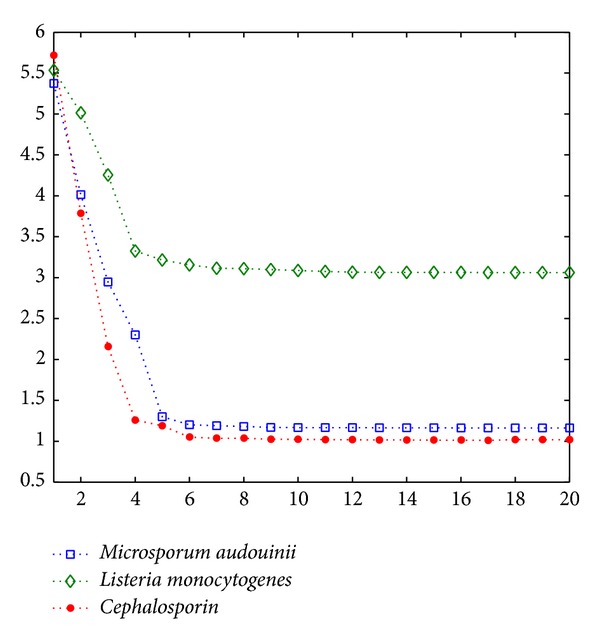
Entropy calculation using Mic, Listeria, and Ceph.

**Figure 6:**
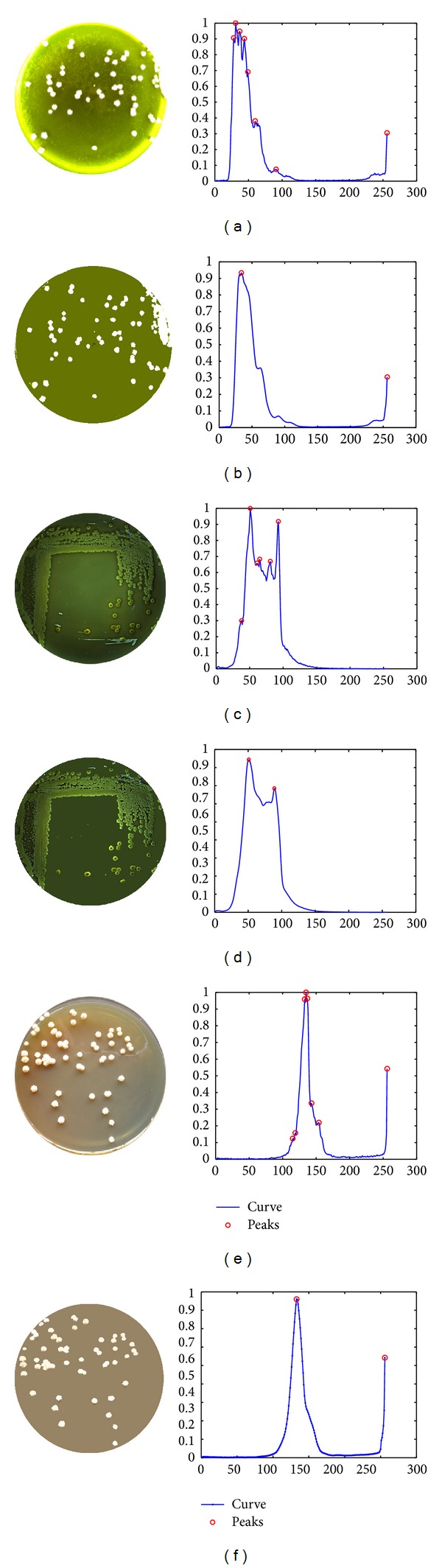
Colony imaging and morphological feature extract.

In image denoising, classical low-passing filters can suppress high frequency noise [24]. However, it is hard for them to preserve the edges of images due to the mixture in some frequency bands. Here, we use energy density spectrums to illustrate the outcomes of different filters [25, 26]. In Figure 4(a), this is the energy spectrum of the original image, where yellow-orange indicates the major energy of symbol “+” and this area is contaminated by the background noise (i.e., blue and green areas).  Figure 4(b) shows the outcome of a low-pass filter, where only the central area of the symbol is kept but the edges of symbol “+” are mixed with the background. In Figure 4(c), based on mean shift, it is clear that the central area of the symbol is outstanding and the edges are also kept. Traditional low-pass filters have good performance on image smoothing but also affect the edge details. In Figure 5 we observe that, after 5 iterations, entropy can reach a stable value, and meanwhile the mean shift iteration automatically stops. Listeria colony entropy is different from the other two. Second row in Figure 6 is Listeria colony. This is because of the cluster colony and agar color is approximated with colony. Figure 6 shows different kinds of colony. The first column shows three different cultures:* Microsporum audouinii*,* Listeria monocytogenes*, and* Cephalosporin*. During the development of cultures, their biochemical reactions appear to be significantly different. As a result, the histograms of cultures in drop-in bright-filed illumination may accompany a number of noisy peaks, illustrated on the second column. Using mean shift based filtering algorithms, we can remove the irregular backgrounds and hence reduce noisy peaks. The result of using mean shift is shown on the 3rd column. Furthermore, we apply an adaptive thresholding based peak searching approach in order to detect two peaks, which indicate the features of cultures. This results in the 4th column.

The performance of mean shift filtering can be measured with mean square error (MSE):

(24) $$\begin{array}{c} \text{MSE}=\frac{1}{BG}\overset{B-1}{\underset{x=0}{\sum }}\overset{G-1}{\underset{y=0}{\sum }}\left[{\left(I^{\prime }\left(x,y\right)-I\left(x,y\right)\right)}^{2}\right], \end{array}$$

where *I*′(*x*, *y*) is denoted by the filtered output image and *I*(*x*, *y*) is denoted by the original input image; Table 1 shows the performance of the three kinds of filtering approach.

After extracting the characteristic peaks, we apply the ULSSVM classifier to the data for classification. We now evaluate the performance of our ULSSVM classifier against the classical SVM [27], LSSVM [28], and fuzzy SVM [29] using 400 colony images, where 150 samples belong to class 1, 50 samples belong to class 2, 100 belong to class 3, and the remainder belong to class 4. We randomly select 300 samples as the training set and the remaining 100 samples are considered as the testing set. There are 130 samples labeled as the unascertained numbers, and half of them are set as training samples.  Table 2 shows the values corresponding to the unascertained information.  Table 3 shows the classification results. In Table 3, the experimental results demonstrate that the ULSSVM effectively improves the performance of classification.

**Figure 7:**
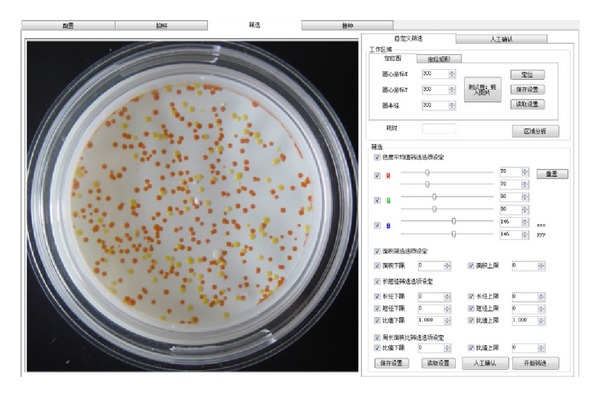
Interface of colony screening.

**Figure 8:**
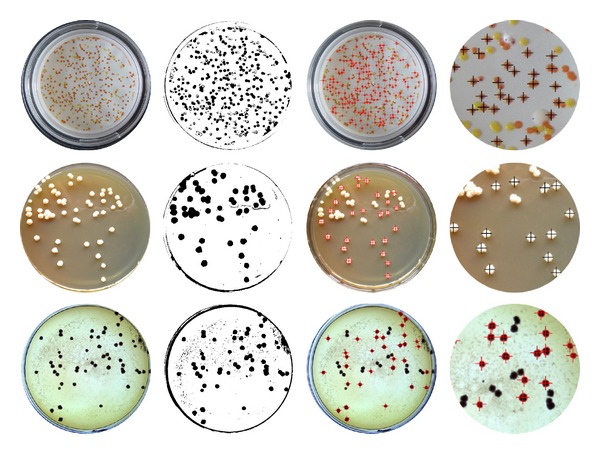
Adaptive colony segmentation based on morphological features.

We carry on screening colony using the ULSSVM classifier.  Figure 7 shows the interface of colony screening.  Figure 8 is adaptive colony segmentation. The segmentation outcomes of two different colony picking methods are as follows: the first row shows the colony with homogeneous medium and its segmentation results using region growing, and the next two rows show the colony with inhomogeneous medium and the corresponding segmentation results using thresholding. The first column is original images, the second column is image process based on our approach, the third column is the identification results, and the fourth column is the local zooming of the screening. Meanwhile, we calculate the time consumption of the colony screen. The process of using the thresholding method took 2.57 s and the process of using the region growing method took 8.61 seconds.

## 5. Conclusions

In this paper, we have deployed an approach to perform adaptive colony segmentation in unstructured environments using feature extraction and selection in an intelligence classifier. We used the intensity histogram peaks as features. To properly determine the importance of the extracted features for colony classification, we used an unascertained theory based LSSVM classification algorithm. Experimental results show that this new approach had better performance than other state-of-the-art techniques in terms of accuracy and speed. This approach works well for adaptive colony segmentation, whilst optimizing the time consumption of colony picking.

## Figures and Tables

**Algorithm 1:**
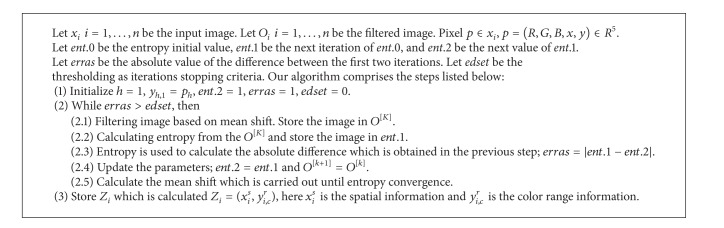
Entropy based mean shift filtering algorithm.

**Algorithm 2:**
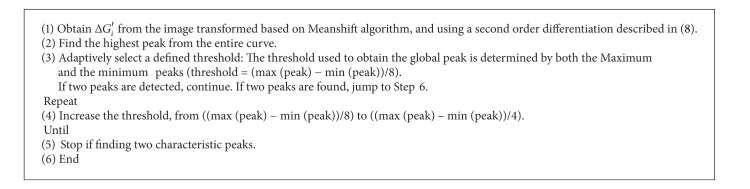
The proposed adaptive quickly peaks detection.

**Table 1:** The filtering performance of three filtering algorithms.

Performance comparison	MSE		
	*α* = 0.1	*α* = 0.3	*α* = 0.5
Median filter	38.541	47.259	85.3254
Fuzzy filter	36.573	49.694	82.3651
Mean shift filter	33.258	40.258	79.6938

**Table 2:** The value corresponding to the unascertained information.

$f_{1}(y)=\{\begin{array}{cc} \begin{array}{c} 0.4\\ 0.3\\ 0.2\\ 0.1 \end{array} & \begin{array}{c} y=1\\ y=2\\ y=3\\ y=4 \end{array} \end{array}$	$f_{2}(y)=\{\begin{array}{cc} \begin{array}{c} 0.3\\ 0.5\\ 0.2\\ 0 \end{array} & \begin{array}{c} y=1\\ y=2\\ y=3\\ y=4 \end{array} \end{array}$	$f_{3}(y)=\{\begin{array}{cc} \begin{array}{c} 0\\ 0.5\\ 0.1\\ 0.4 \end{array} & \begin{array}{c} y=1\\ y=2\\ y=3\\ y=4 \end{array} \end{array}$	$f_{4}(y)=\{\begin{array}{cc} \begin{array}{c} 0.7\\ 0.3\\ 0\\ 0 \end{array} & \begin{array}{c} y=1\\ y=2\\ y=3\\ y=4 \end{array} \end{array}$	$f_{5}(y)=\{\begin{array}{cc} \begin{array}{c} 0.4\\ 0\\ 0.1\\ 0.5 \end{array} & \begin{array}{c} y=1\\ y=2\\ y=3\\ y=4 \end{array} \end{array}$

$f_{6}(y)=\{\begin{array}{cc} \begin{array}{c} 0.5\\ 0.1\\ 0.4\\ 0 \end{array} & \begin{array}{c} y=1\\ y=2\\ y=3\\ y=4 \end{array} \end{array}$	$f_{7}(y)=\{\begin{array}{cc} \begin{array}{c} 0.1\\ 0.4\\ 0.3\\ 0.2 \end{array} & \begin{array}{c} y=1\\ y=2\\ y=3\\ y=4 \end{array} \end{array}$	$f_{8}(y)=\{\begin{array}{cc} \begin{array}{c} 0.7\\ 0.2\\ 0\\ 0.1 \end{array} & \begin{array}{c} y=1\\ y=2\\ y=3\\ y=4 \end{array} \end{array}$	$f_{9}(y)=\{\begin{array}{cc} \begin{array}{c} 0.5\\ 0.1\\ 0.3\\ 0 \end{array} & \begin{array}{c} y=1\\ y=2\\ y=3\\ y=4 \end{array} \end{array}$	$f_{10}(y)=\{\begin{array}{cc} \begin{array}{c} 0.2\\ 0.3\\ 0.1\\ 0.4 \end{array} & \begin{array}{c} y=1\\ y=2\\ y=3\\ y=4 \end{array} \end{array}$

**Table 3:** Classifiers' experimental results.

Classifier	SVM	FSVM	LSSVM	ULSSVM
Accuracy	75.9%	84.6%	73.2%	**92.7%**
Training speed/s	12.1 s	13.7 s	5.1 s	**2.7 s**
